# The Association between Socioeconomic Status, Smoking, and Chronic Disease in Inner Mongolia in Northern China

**DOI:** 10.3390/ijerph16020169

**Published:** 2019-01-09

**Authors:** Xuemei Wang, Ting Zhang, Jing Wu, Shaohua Yin, Xi Nan, Maolin Du, Aiping Liu, Peiyu Wang

**Affiliations:** 1 Department of Nutrition and Food Hygiene, School of Public Health, Peking University Health Science Center, Beijing 100191, China; wangxm@immu.edu.cn; 2 Department of Health Statistics, School of Public Health, Inner Mongolia Medical University, Hohhot 010110, China; nx1994ynx@163.com (X.N.); dumaolin@immu.edu.cn (M.D.); 3 Department of Social Medicine and Health Education, School of Public Health, Peking University Health Science Center, Beijing 100191, China; tingting5063@163.com; 4 National Center for Chronic and Non-Communicable Disease Control and Prevention, Chinese Center for Disease Control and Prevention, Beijing 100050, China; wujing@chinacdc.cn; 5 Department of Reproductive and Child Health, School of Public Health, Peking University Health Science Center, Beijing 100191, China; yinsh@pku.edu.cn

**Keywords:** socioeconomic status (SES), smoking, chronic disease, interaction

## Abstract

The interactive associations of socioeconomic status (SES) and smoking with chronic disease were investigated with a view to expanding the evidence to inform tobacco policies and interventions in Northern China. The fifth NHSS (National Health Service Survey) 2013 in Inner Mongolia was a population-based survey of national residents, aged 15 years and older, in which multi-stage stratified cluster sampling methods were used to survey 13,554 residents. The SES was measured by scores derived from levels of education level and household annual income. Multivariate logistic regression models were performed to determine the association between SES, smoking, and chronic disease adjusted by confounders. Three thousand nine hundred and thirty-seven residents (32.29%) were identified as current smokers and 3520 residents (26.01%) had been diagnosed with chronic diseases. In the males, former smoking with low SES had the highest risk of one chronic disease, with an odds ratio (OR) of 2.505 (95% confidence interval [95% CI] (OR = 2.505, 95% CI: 1.635–3.837) or multiple chronic diseases (OR = 2.631, 95% CI: 1.321–5.243). In the females, current smoking with low SES had the highest risk of one chronic disease (OR = 3.044, 95% CI: 2.158–4.292). The conclusion of this study was that residents with combined ever-smoking and low SES deserved more attention in the prevention and control of chronic disease.

## 1. Introduction

Smoking is known to result in a wide range of negative health consequences [1,2]. It is estimated that, by 2025, the number of smokers worldwide will increase to 1.6 billion—with 80% of those living in developing countries. Furthermore, smoking is set to become the leading cause of death worldwide by 2020 [3]. Smoking has also emerged as one of the most serious public health problems in China [4]. Meanwhile, there is evidence that smoking is associated with many individual-level socioeconomic indicators, such as income, education, and occupation, and it is more frequent among socioeconomically disadvantaged people [5]. The association between smoking and poverty is apparent at all levels [6]. First of all, the risk that a young person will begin smoking is greater in less privileged groups [7]. Additionally, quit rates are lower in the poorest groups and for those living in socially disadvantaged areas [8]. Furthermore, poor people tend to smoke more both in terms of prevalence and consumption [9], and the burden of smoking-related disease is typically concentrated among socially disadvantaged groups in particular [1,2]. The risk of dying from smoking is significantly higher in the lowest socioeconomic groups compared to the highest socioeconomic groups. Nevertheless, socioeconomic status was not usually considered a direct risk factor related to chronic diseases [10]. It was even suggested that, in China, with the development of the economy, the higher socioeconomic status, the higher the risk for chronic diseases, due to people adopting a Westernized diet [11]. One study indicated that without meaningful efforts at encouraging smoking cessation, one third to half of all male smokers in China could die by 2030 [12]—with even higher rates of mortality among the socioeconomically disadvantaged population. While the chronic disease burden among the rural and urban socioeconomic disadvantaged population is a major policy concern in China, socioeconomic status itself is closely associated with chronic disease risk both in China and other developing countries [13,14,15]. One contributor to the stubbornly high rates of chronic disease in China is tobacco use, which is an important risk factor for chronic diseases such as cancer, cardiovascular disease, diabetes, and chronic lung disease [16]. Targeted interventions have aimed to reduce smoking rates among socioeconomically deprived groups, which have been proven to be effective at increasing life expectancy and improving health status [17]. With a view to expanding the existing evidence in order to develop future public health interventions in Northern China, we analyzed the interactive association between socioeconomic status, smoking, and chronic disease in Inner Mongolia using data from the fifth National Health Service Survey.

## 2. Materials and Methods

### 2.1. NHSS Methods in Inner Mongolia

As part of data collection for the fifth NHSS in 2013 in Inner Mongolia (an autonomous region in northern China), multi-stage stratified cluster sampling was used to ensure a representative cross-section of the population aged ≥15 years. The NHSS was approved by the National Commission Health and Family Planning China to occur every fifth year since 1993. Until now, the NHSS has been conducted five times (1993, 1998, 2001, 2003, 2008, and 2013). Samples were distributed among agricultural and pastoral areas, forested regions, border areas, mineral-rich areas dominated by mining industries, areas with high socioeconomic deprivation, and regions with significant ethnic minority populations. Sampling took place in 65 counties and Sumu (a type of country-level administrative division in Inner Mongolia) distributed among 54 counties. Extracting Two Gacha (villages) were extracted from each district (banner), and then 33 families were chosen from each district (banner). 

### 2.2. Ethics Approval and Consent to Participate

This survey was organized by the Inner Mongolia government. In this survey, there were no treatments, blood drawings, or other interventions that could impact the health of participators. Informed consent was obtained by surveyors prior to data collection and personal information was with strict conservation during our analysis process. Thus, no ethical approval was required for our study.

### 2.3. Variable Definitions

The survey included items related to socio-demographic characteristics such as age, sex, level of education, employment status, health behaviors such as smoking and alcohol use, and chronic diseases diagnosed by a doctor, such as hypertension, diabetes, and others. Education was categorized into two levels (middle school level or below, high school or above) and household annual income was categorized into three levels (<30,000 RMB, 30,000~80,000 RMB, >80,000 RMB). The SES was then measured by the above two indicators: education level and household annual income. The SES score was derived from adding the level of education (middle school and below, score of 0; high school and above, score of 1) and household annual income (<30,000 RMB, score of 0; ≥30,000 RMB, score of 1). Respondents who had consumed alcohol within the past 12 months were defined as “drinking” and those who had not consumed alcohol within this period were defined as “not drinking” (the definition of drinking was based on the content of the questionnaire in the health service survey, where the participants were asked ‘‘Did you drink alcohol in the past 12 months?”; if the participant answered “yes”, it was defined as “drinking”, and if the answer was “no”, it was defined as “not drinking”). Meanwhile, “nonsmoking”, “former smoking”, and “current smoking” were defined as those who had never smoked previously, those who had previously smoked but had quit, and those who are currently smoking, respectively. The outcome of this study was self-reported chronic disease. The self-reported chronic disease was defined as one of the following conditions: having been diagnosed with chronic disease by doctors sometime six months prior to completing the survey suffering from chronic disease diagnosed by doctors and having current chronic disease treatments, such as taking medicine, taking physiotherapy, or accepting treatment to control the progress of chronic disease sometime six months prior to completing the survey. Respondents were also categorized into three levels (none, one, and two or above) according to whether they had been medically diagnosed with the following chronic diseases: hypertension, diabetes, cerebrovascular disease, cardiovascular disease, or chronic obstructive pulmonary disease. 

### 2.4. Data Analyses

Firstly, a univariate analysis was performed. We compared smoking, smoking cessation, and non-smoking by age, race/ethnicity, employment status, marital status, settlement, drinking status, level of education, household annual income, and socioeconomic score using chi-squared test for both sexes. Furthermore, we analyzed the relationship between different smoking statuses and socioeconomic score. Chronic disease prevalence was then analyzed by these demographic dimensions, SES score, and smoking status using chi-squared test for both sexes. Variables in the univariate analysis with *p* ≤ 0.10 were included in a multivariable analysis. We then used multiple logistic regression to independently assess the risk of both demographic predictors and socioeconomic status of chronic disease. The interaction between socioeconomic status and smoking on chronic disease was also analyzed. The association of chronic disease with an interaction between socioeconomic status and smoking was tested; the multivariate models were adjusted by age, employment status, body mass index (BMI), and alcohol use for both sexes. All statistical analyses were performed with SPSS software version 19.0 (IBM Corp, Armonk, NY, USA). Statistical significance was determined by *α* < 0.05.

## 3. Results

### 3.1. Smoking Prevalence according to Sex and Socio-Demographic Characteristics

The distribution of smoking status by different socio-demographic characteristics is shown in a supplementary table (please see Table S1 in the supplementary file). Using the 2010 national demographic criteria to standardize, out of a total of 13,554 residents aged 15 years and older, 3937 (32.29%, standardized rate 30.54%) were identified as current smokers and 3520 (26.01%, standardized rate 19.99%) had been diagnosed with chronic diseases. Furthermore, we found significant differences in age, ethnicity, level of education, household annual income, employment status, marital status, settlement, body mass index, and alcohol use according to smoking status in both sexes. Smoking prevalence was highest in the 45–59 years age group among both sexes.

### 3.2. Socioeconomic Status and Smoking Prevalence According to Sex

Of the 13,354 residents, 71.5% had an educational level at the middle school level or below and 35.8% had a household annual income < 30,000 RMB. The scores were added from the educational level and household annual income; the lower the score, the worse the socioeconomic status. Table 1 shows the socioeconomic status and smoking prevalence according to sex. The results of the chi-squared test showed that there was a social gradient association of SES with smoking prevalence in both sexes; the higher current smoking rate or former smoking rate, the lower the SES score (i.e., the worse the socioeconomic status).

### 3.3. Chronic Disease Prevalence According to Sex, Socioeconomic Status, and Socio-Demographic Characteristics

Our results show that the prevalence of residents with one chronic condition or multiple chronic conditions was significantly higher in the lower SES group in both sexes. Socioeconomic status, age, ethnicity, level of education, employment status, marital status, smoking status, body mass index, alcohol use, and household annual income all varied significantly according to the presence of chronic conditions (*p* < 0.05). Furthermore, the prevalence of residents with one chronic condition and multiple chronic conditions increased with age in both sexes (*p* < 0.001) (Table 2).

### 3.4. Predictors of Chronic Conditions by Socioeconomic Status Interaction with Smoking

Table 3 shows an interaction between socioeconomic status and smoking on chronic disease. Compared with high socioeconomic status (SES = 2) and the non-smoking group, each of the other lower socioeconomic statuses with current smoking or former smoking, as well as low socioeconomic status with nonsmoking, displayed a higher risk of diagnosis with one chronic condition in both sexes. After adjusting for other variables, in the males, individuals in former smoking group with low SES had the highest risk of one chronic disease, with an odds ratio ( OR ) of 2.505 (95% confidence interval [95% CI]: 1.635–3.837) (OR = 2.505, 95% CI: 1.635–3.837) or multiple chronic diseases (OR = 2.631, 95% CI: 1.321–5.243). In the females, individuals in the current smoking group with low SES had the highest risk of one chronic disease (OR = 3.044, 95% CI: 2.158–4.292). 

### 3.5. Simple Effect of Chronic Disease Risk at Different Socioeconomic Status or Smoking Status

**Figure 1:**
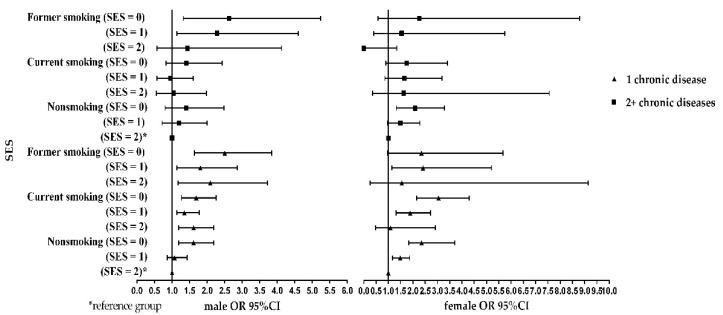
Association of SES with chronic disease per status of smoking, stratified by sex.

**Figure 2:**
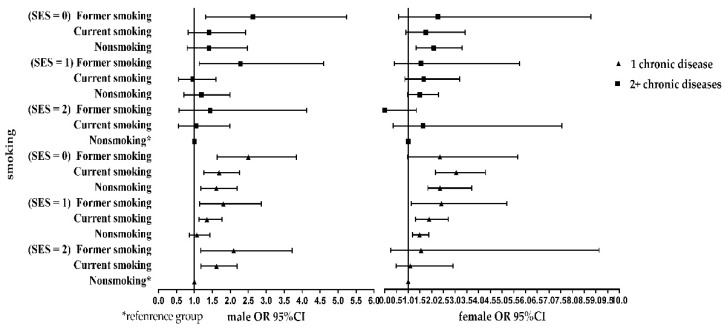
Association of smoking status with chronic disease per SES, stratified by sex.

Figure 1 and Figure 2 show the simple effect of socioeconomic status, smoking status on chronic disease.

At the same smoking status level, compared with high socioeconomic status, low socioeconomic status had a higher risk of one or multiple chronic diseases in both sexes. At the same socioeconomic status level, compared with nonsmokers, the risk of suffering from one or multiple chronic diseases was higher with former smoking among the males. However, the risk of diagnosis with multiple chronic diseases was higher among females in the current smoking group than the female nonsmokers. Perhaps the socioeconomic status is the most influential factor for females suffering from chronic diseases. 

## 4. Discussion

Prevalence of smoking was higher among the lower socioeconomic status, suggesting that, as in the United States, low socioeconomic status is a major independent determinant factor of smoking in adults [18]. Several studies [19,20] have shown that the rate of smoking was 21.6% in Shanghai and 22.92% in Zhejiang province, respectively, which were significantly lower than our study (30.54%). Additionally, previous studies [21,22] have shown that smoking is associated with low levels of education, unemployment, and poor marital status; our results also identify the Han ethnicity, poor marital status, and alcohol use as predictors of smoking among economically disadvantaged residents in both sexes. Other studies [23] have indicated that the rate of chronic disease in Jiangxi province was 7.9%, while in our study the rate of chronic disease was 19.99%, which is significantly higher than in Jiangxi. Our results also identify strong two-way associations between socioeconomic status and chronic disease, as in previous studies [24,25,26,27,28], suggesting the presence of a vicious cycle in such disadvantaged rural communities in which poverty-related diseases intensify the effects of low socioeconomic status and contribute to its persistence [29]. Socioeconomic factors such as level of education, income, and occupational status have been found to be strongly associated with the prevalence of multiple chronic conditions in both developed and developing countries [30,31,32,33,34]. A recent multi-country study [35] found that the prevalence of multiple chronic conditions increased with age and was higher among females and respondents with a lower level of education, which was consistent with our own results. Furthermore, the high prevalence of chronic conditions in the elder age group may have been a result of an age-related decline in physical functioning and poor health behaviors. Residents with low levels of education have been shown to be more likely to engage in heavy manual labor, experience a higher degree of work-related stress, and exhibit poor health behaviors and have less awareness of chronic diseases and their prevention [36]. As a previous study [37] has shown in a developing country context, alcohol and tobacco use are major determinants of chronic disease prevalence, particularly among economically disadvantaged communities. Specifically, exposure to nicotine, tar, and other harmful substances found in cigarettes and alcohol has been linked with over 60 chronic diseases and types of trauma [38]; in addition, long-term heavy alcohol use is widely known to accelerate the development of chronic disease through damage to the heart, liver, blood vessels, and other organs.

There was an additive interaction between socioeconomic status and smoking. When compared with high socioeconomic status (SES = 2) and non-smoking, the risk of chronic conditions in each of the lower socioeconomic statuses with current smoking and former smoking was higher in both sexes. Residents with low socioeconomic status are often faced with unfavorable living conditions, higher rates of divorce and unemployment, exposure to adverse working environments, elevated levels of work stress, and a higher acceptance of smoking in the community—all of which are associated with higher rates of smoking, which also greatly increases the risk of chronic disease for these people [39,40]. Meanwhile, male residents of high socioeconomic status who are current smokers are more likely to have high pressured jobs or busy lives, which has a serious impact on health, increasing the likelihood of developing a higher risk of chronic disease, thus promoting smoking cessation in turn [41,42].

Our findings also showed that the majority of smokers (68%) in our sample began smoking from 16 to 24 years of age—suggesting that uptake of smoking in adolescence may be an emerging trend in Inner Mongolia, as in other developing regions [43]. This is especially concerning in light of a previous study that has shown that residents who begin smoking at younger ages tend to consume more cigarettes and are less likely to quit [44]. Known determinants of smoking uptake in young adults include influence from family members, stress resulting from work or studies, and acceptance of smoking in the wider community [45]. Given that adolescents with poor awareness of the health implications are more likely to smoke [46], tobacco control interventions should focus on improving health knowledge while also addressing causes of stress and attitudes towards smoking in the community to maximize their effectiveness in preventing smoking among young adults [47].

Our findings also showed a significant difference (65.8% vs. 20.7%) in the prevalence of smoking between those categorized into the drinking versus not drinking groups. Low-income residents of Inner Mongolia are typically concentrated in rural areas and are engaged in heavy manual work in agriculture, resulting in an elevated exposure to work stress and adverse working conditions. These factors, alongside traditional cultural norms surrounding drinking, are likely to have contributed to the high levels of alcohol use in our study population. Given that drinking and smoking show strong clustering, and that drinking, even in moderation, has been shown to impede smoking cessation [48], we recommend that future health information programs attempt to address both health behaviors simultaneously [49].

Additionally, our results showed that the prevalence of former smoking was higher among those in lower socioeconomic status groups. Our results also revealed that the proportion of former smokers is higher among those respondents diagnosed with one or multiple chronic conditions than non-smokers and current smokers, especially in the males. One explanation may be that smokers who are diagnosed with a chronic disease may be more motivated to quit and receive greater support to do so from doctors and family members. This hypothesis is supported by a previous study that indicated that low SES smokers are likely or more likely than other smokers to attempt to quit following a chronic disease diagnosis [50]. This may represent an attempt to minimize the potential costs associated with chronic disease to themselves and family members. However, as shown in previous studies, females are often less able to quit smoking than males. The explanation may be that male smokers smoke for the reinforcing drug effect whereas female smokers smoke for other reasons, such as mood regulation and cue reactivity; additionally, females are more likely to acknowledge perceived risks of quit smoking (e.g., weight gain, negative effect) [51,52].

## 5. Conclusions

In conclusion, the present study highlights the interactive association of socioeconomic status and smoking with chronic disease—in addition to the scale of the challenge posed by the high chronic disease burden among low SES residents. At the same time, the high rate of chronic disease among high SES residents who are smokers, and who are more likely to have high pressured jobs or busy lives, should also be given sufficient attention. Future tobacco control interventions, particularly health education programs, should be targeted according to groups with different socioeconomic statuses. Special attention should be paid for disadvantaged communities (including adolescents and old people) and highly pressured populations, thereby challenging existing social attitudes around smoking and reducing the tobacco-related disease burden [53]. Furthermore, gender differences should be considered, and measures aimed at preventing chronic disease and reducing poverty should play a key role in improving the quality of life among residents in Inner Mongolia [29]—particularly among the low SES residents. 

## Figures and Tables

**Table 1:** Socioeconomic status and smoking prevalence according to sex.

Variable	Male (*n* = 6721)					Female (*n* = 6814)				
	Non-Smoking	Former Smoking	Current Smoking	*χ* ^2^	*p*-Value	Non-Smoking	Former Smoking	Current Smoking	*χ* ^2^	*p*-Value
	*n* (%)	*n* (%)	*n* (%)			*n* (%)	*n* (%)	*n* (%)		
**SES score**				89.806	<0.001 ^^^				102.084	<0.001 ^^^
2	747 (49.73)	78 (5.19)	677 (45.07)			1158 (95.00)	12 (0.98)	49 (4.02)		
1	1138 (40.10)	149 (5.25)	1551 (54.65)			2445 (87.48)	45 (1.61)	305 (10.91)		
0	684 (35.76)	169 (8.83)	1060 (55.41)			1597 (83.09)	30 (1.56)	295 (15.35)		

Scores were added from the following: middle school education level and below (1 score), income < 30,000 RMB (1 score); ^^^ *p* < 0.001. SES: socioeconomic status.

**Table 2:** Socioeconomic status and socio-demographic characteristics of residents according to chronic conditions for male and female separately.

Variable	Male					Female				
	0	1	2+	*χ* ^2^	*p*-Value	0	1	2+	*χ* ^2^	*p*-Value
	*n* (%)	*n* (%)	*n* (%)			*n* (%)	*n* (%)	*n* (%)		
**SES score**				38.158	<0.001 ^^^				199.854	<0.001 ^^^
2	1348 (81.06)	264 (15.87)	51 (3.07)			1287 (87.02)	152 (10.28)	40 (2.70)		
1	2442 (80.20)	477 (15.67)	126 (4.14)			2455 (76.96)	567 (17.77)	168 (5.27)		
0	1489 (74.08)	412 (20.50)	109 (5.42)			1427 (66.78)	556 (26.02)	154 (7.21)		
**Age**				998.204	<0.001 ^^^				1474.931	<0.001 ^^^
<45	2807 (94.07)	165 (5.53)	12 (0.40)			2858 (95.71)	121 (4.05)	7 (0.23)		
45~59	1618 (73.71)	487 (22.19)	90 (4.10)			1581 (70.45)	544 (24.24)	119 (5.30)		
60~	857 (55.58)	501 (32.49)	184 (11.93)			737 (46.53)	611 (38.57)	236 (14.90)		
**Ethnicity**				8.638	0.071				27.531	<0.001 ^^^
Han	4214 (78.50)	918 (17.10)	236 (4.40)			3977 (74.77)	1045 (19.65)	297 (5.58)		
Mongolian	823 (80.53)	165 (16.14)	34 (3.33)			897 (82.07)	157 (14.36)	39 (3.57)		
Others	231 (73.33)	68 (21.59)	16 (5.08)			289 (75.06)	72 (18.70)	24 (6.23)		
**Employment status**				543.707	<0.001 ^^^				568.705	<0.001 ^^^
Unemployed	477 (69.03)	150 (21.71)	64 (9.26)			888 (64.44)	362 (26.27)	128 (9.29)		
Retired	396 (51.10)	273 (35.23)	106 (13.68)			441 (51.70)	296 (34.70)	116 (13.60)		
Employed	4404 (83.90)	729 (13.89)	116 (2.21)			3836 (83.94)	616 (13.48)	118 (2.58)		
**Marital status**				257.328	<0.001 ^^^				437.693	<0.001 ^^^
Single	899 (97.19)	24 (2.59)	2 (0.22)			614 (96.69)	14 (2.20)	7 (1.10)		
Widowed or divorced	222 (64.72)	86 (25.07)	35 (10.20)			318 (49.00)	232 (35.75)	99 (15.25)		
Married	4156 (76.28)	1043 (19.14)	249 (4.57)			4235 (76.71)	1030 (18.66)	256 (4.64)		
**Urban/Rural**				10.144	0.006 ^&^				13.912	<0.001 ^^^
Urban	1656 (76.60)	394 (18.22)	112 (5.18)			1740 (75.69)	406 (17.66)	153 (6.66)		
Rural	3626 (79.53)	759 (16.65)	174 (3.82)			3436 (76.10)	870 (19.27)	209 (4.63)		
**Smoking status**				102.811	<0.001 ^^^				20.759	<0.001 ^^^
Current smoking	2609 (79.35)	570 (17.34)	109 (3.32)			458 (70.57)	157 (24.19)	34 (5.24)		
Former smoking	237 (59.85)	117 (29.55)	42 (10.61)			56 (64.37)	25 (28.74)	6 (6.90)		
Non-smoking	2059(80.12)	397 (15.45)	114 (4.44)			3991 (76.72)	941 (18.09)	270 (5.19)		
**Alcohol use**				16.452	<0.001 ^^^				5.398	0.067
Yes	2314 (79.33)	512 (17.55)	91 (3.12)			261 (80.06)	56 (17.18)	9 (2.76)		
No	2968 (78.02)	641 (16.85)	195 (5.13)			4914 (75.75)	1220 (18.81)	353 (5.44)		
**BMI**				124.722	<0.001 ^^^				273.888	<0.001 ^^^
<24	3245 (82.89)	540 (13.79)	130 (3.32)			3553 (81.92)	623 (14.36)	161 (3.71)		
24~28	1614 (74.52)	441 (20.36)	111 (5.12)			1320 (68.11)	472 (24.36)	146 (7.53)		
≥28	408 (65.81)	169 (27.26)	43 (6.94)			286 (55.21)	180 (34.75)	52 (10.04)		
**Education level**				14.583	<0.001 ^^^				167.937	<0.001 ^^^
Middle school and below	3621 (77.36)	843 (18.01)	217 (4.64)			3602 (71.92)	1095 (21.87)	311 (6.21)		
High school and above	1658 (81.39)	310 (15.22)	69 (3.39)			1567 (87.15)	180 (10.01)	51 (2.84)		
**Household annual income**				24.907	<0.001 ^^^				90.369	<0.001 ^^^
Low	1800 (75.47)	458 (19.20)	127 (5.32)			1709 (69.53)	584 (23.76)	165 (6.71)		
Middle	2817 (80.05)	575 (16.34)	127 (3.61)			2810 (79.04)	581 (16.34)	164 (4.61)		
High	665 (81.40)	120 (14.69)	32 (3.92)			657 (82.02)	111 (13.86)	33 (4.12)		

^&^*p* < 0.05; ^^^ *p* < 0.001.

**Table 3:** Results from a multinomial logistic regression analysis of socioeconomic status interaction with smoking status on the number of chronic conditions diagnosed among residents in Inner Mongolia for male and female separately.

Variable	Male		Female	
	1	2+	1	2+
	OR (95% CI)	OR (95% CI)	OR (95% CI)	OR (95% CI)
**Univariate model ^$^**				
(SES = 2) Nonsmoking	1	1	1	1
(SES = 1) Nonsmoking	1.185 (0.972−1.557)	1.332 (0.822−2.169)	1.883 (1.513−2.344) ^*^	2.052 (1.381−3.453) ^*^
(SES = 0) Nonsmoking	1.825 (1.371−2.431) ^*^	1.756 (1.449−2.944) ^*^	3.273 (2.619−4.098) ^*^	3.344 (2.238−4.995) ^*^
(SES = 2) Current smoking	1.500 (1.119−2.211) ^*^	0.806 (0.438−1.488)	1.026 (0.398−2.646)	1.501 (0.348−6.475)
(SES = 1) Current smoking	1.281 (0.991−1.656)	0.819 (0.499−1.346)	2.355 (1.677−3.349) ^*^	2.222 (1.199−4.125) ^*^
(SES = 0) Current smoking	1.704 (1.387−2.223) ^*^	1.436 (0.881−2.341)	4.107 (2.995−5.631) ^*^	2.669 (1.436−4.962) ^*^
(SES = 2) Former smoking	3.267 (1.915−5.574) ^*^	2.460 (0.955−6.691)	2.874 (0.767−14.767)	0.000 (0.000−3.210)
(SES = 1) Former smoking	2.828 (1.834−4.359) ^*^	4.718 (2.489−8.944) ^*^	3.866 (1.955−7.643) ^*^	3.261 (0.944−11.268)
(SES = 0) Former smoking	3.752 (2.521−5.584) ^*^	4.773 (2.544−8.956) ^*^	4.312 (1.894−9.817) ^*^	5.255 (1.473−18.749) ^*^
**Multivariate model ^$#^**				
(SES = 2) Nonsmoking	1	1	1	1
(SES = 1) Nonsmoking	1.072 (0.866−1.429)	1.194 (0.715−1.996)	1.485 (1.177−1.873) ^*^	1.493 (0.975−2.287)
(SES = 0) Nonsmoking	1.613 (1.185−2.194) ^*^	1.410 (0.802−2.481)	2.355 (1.842−3.713) ^*^	2.092 (1.329−3.292) ^*^
(SES = 2) Current smoking	1.615 (1.188−2.196) ^*^	1.047 (0.553−1.985)	1.089 (0.487−2.913)	1.628 (0.351−7.554)
(SES = 1) Current smoking	1.352 (1.133−1.771) ^*^	0.952 (0.564−1.609)	1.891 (1.316−2.715) ^*^	1.656 (0.861−3.193)
(SES = 0) Current smoking	1.689 (1.269−2.257) ^*^	1.413 (0.823−2.428)	3.044 (2.158−4.292) ^*^	1.750 (0.896−3.418)
(SES = 2) Former smoking	2.094 (1.176−3.728) ^*^	1.443 (0.575−4.127)	1.548 (0.262−9.137)	0.000 (0.000−1.350)
(SES = 1) Former smoking	1.808 (1.141−2.863) ^*^	2.287 (1.138−4.596) ^*^	2.414 (1.143−5.194) ^*^	1.541 (0.413−5.754)
(SES = 0) Former smoking	2.505 (1.635−3.837) ^*^	2.631 (1.321−5.243) ^*^	2.350 (0.973−5.676)	2.268 (0.585−8.789)

^$^ With socioeconomic status and smoking status as the independent variable and residents with SES score of 2 and nonsmoking as the reference group. ^#^ Adjusted for age, sex, employment status, BMI and alcohol use. ^*^ *p* < 0.05.

## Supplementary Materials

The following are available online at http://www.mdpi.com/1660-4601/16/2/169/s1, Table S1: The distribution of smoking status according to sex and socio-demographics characteristics.
