# Supplementary material for: The Association between Socioeconomic Status, Smoking, and Chronic Disease in Inner Mongolia in Northern China
Source: Int J Environ Res Public Health. 2019 Jan 9;16(2):169. doi: 10.3390/ijerph16020169 (PMC6352126; doi:10.3390/ijerph16020169)
Supplement: Supplementary file 1 [file ijerph-16-00169-s001.pdf]

Table S1. The distribution of smoking status according to sex and socio-demographics characteristics.

Variable	Male (n = 6721)					Female (n = 6814)				
	Nonsmoking n (%)	Former smoking n (%)	Current smoking n (%)	$\chi 2$	p-value	Nonsmoking n (%)	Former smoking n (%)	Current smoking n (%)	$\chi 2$	p-value
**Age**				318.191	<0.001^				136.264	<0.001^
<45	1283 (47.01)	77 (2.82)	1369 (50.16)			2376 (92.96)	18 (0.70)	162 (6.34)		
45~59	705 (33.68)	108 (5.16)	1280 (61.16)			1663 (84.16)	26 (1.32)	287 (14.52)		
60~	582 (40.64)	211 (14.73)	639 (44.62)			1163 (82.72)	43 (3.06)	200 (14.22)		
**Ethnicity**				48.545	<0.001^				5.758	<0.001^
Han	1953 (39.11)	340 (6.81)	2700 (54.08)			4038 (87.33)	74 (1.60)	512 (11.07)		
Mongolian	492 (50.57)	38 (3.91)	443 (45.53)			867 (87.75)	10 (1.01)	111 (11.23)		
Others	118 (42.75)	18 (6.52)	140 (50.72)			287 (91.11)	3 (0.95)	25 (7.94)		
**Education level**				79.754	<0.001^				96.489	<0.001^
Middle school and below	1654 (37.50)	293 (6.64)	2464 (55.86)			3792 (85.21)	71 (1.60)	587 (13.19)		
High school and above	915 (49.67)	103 (5.59)	824 (44.73)			1408 (94.75)	16 (1.08)	62 (4.17)		
**Household annual income**				45.224	<0.001^				39.864	<0.001^
Low	852 (37.82)	194 (8.61)	1207 (53.57)			1847 (84.38)	34 (1.55)	308 (14.07)		
Middle	1421 (43.75)	169 (5.20)	1658 (51.05)			2725 (88.97)	45 (1.47)	293 (9.57)		
High	297 (39.44)	33 (4.38)	423 (56.18)			630 (91.84)	8 (1.17)	48 (7.00)		
**Employment status**				125.997	<0.001^				16.435	0.002&
Unemployed	242 (38.23)	76 (12.01)	315 (49.76)			1025 (84.78)	21 (1.74)	163 (13.48)		
Retired	320 (45.65)	90 (12.84)	291 (41.51)			609 (88.26)	16 (2.32)	65 (9.42)		
Employed	2006 (40.80)	230 (4.68)	2681 (54.53)			3562 (88.34)	50 (1.24)	420 (10.42)		
**Marital status**				237.585	<0.001^				35.463	<0.001^
Single	528 (65.67)	19 (2.36)	257 (31.97)			486 (94.92)	2 (0.39)	24 (4.69)		
Widowed or divorced	115 (35.71)	31 (9.63)	176 (54.66)			483 (83.42)	13 (2.25)	83 (14.34)		
Married	1926 (37.57)	346 (6.75)	2855 (55.69)			4231 (87.33)	72 (1.49)	542 (11.19)		
**Settlement**				20.947	<0.001^				48.131	<0.001^
Urban	875 (45.24)	105 (5.43)	954 (49.33)			1732 (91.93)	21 (1.11)	131 (6.95)		
Rural	1695 (39.24)	291 (6.74)	2334 (54.03)			3470 (85.59)	66 (1.63)	518 (12.78)		
**Alcohol use**				632.716	<0.001^				125.249	<0.001^
Yes	691 (24.52)	162 (5.75)	1965 (69.73)			209 (67.86)	6 (1.95)	93 (30.19)		
No	1879 (54.69)	234 (6.81)	1323 (38.50)			4992 (88.68)	81 (1.44)	556 (9.88)		
**BMI***				16.360	0.003&				13.507	0.009&
<24	1440 (39.39)	220 (6.02)	1996 (54.60)			3263 (86.53)	61 (1.62)	447 (11.85)		
24~28	887 (44.13)	130 (6.47)	993 (49.40)			1521 (89.42)	23 (1.35)	157 (9.23)		
≥28	238 (41.50)	44 (7.70)	292 (50.90)			408 (89.90)	2 (0.40)	44 (9.70)		

*Body mass index

& $p < 0.05$

^ $p < 0.001$
